# 8q22.2q22.3 Microdeletion Syndrome Associated with Hearing Loss and Intractable Epilepsy

**DOI:** 10.1155/2019/7608348

**Published:** 2019-01-10

**Authors:** Alejandra Rincon, Paola Paez-Rojas, Fernando Suárez-Obando

**Affiliations:** ^1^Instituto de Genética Humana, Facultad de Medicina, Pontificia Universidad Javeriana, Colombia; ^2^Javesalud, Colombia

## Abstract

8q22.2q22.3 microdeletion syndrome has been described in only seven patients. We present a new case from Colombia. The characteristics of this condition are developmental delay, microcephaly, seizures, and typical facial dysmorphism. We discuss the clinical phenotype of the patient presenting relevant findings like hearing loss and severe epilepsy and the possible relations between the phenotype and the genes involved in the microdeletion. We describe a female with developmental delay, microcephaly, epilepsy, severe short stature, impaired speech, facial dysmorphism, and congenital deafness. A minimal/maximal deletion of 5.238 Mb and 5.374Mb, respectively, at 8q22.2q22.3 was diagnosed using a genome-wide array. The clinical phenotype is similar to the others seven patients previously reported; however, the severity of epilepsy and the concomitant hearing loss is remarkable, characteristics previously observed independently in only two patients. The KCNS2 gene is located in the deleted regions (8q22.2). Therefore it is a possible candidate for explaining the complex neurologic phenotype.

## 1. Background

Developmental delay and intellectual disability (DD/ID) associated with abnormal phenotype are common indications for genetic referral [1]. In developing countries like Colombia, the diagnostic approach is usually based on clinical approach, neurological assessment, conventional karyotyping, and screening tests for metabolic diseases. However, until recently the clinical availability of MLPA (multiplex ligation-dependent probe amplification) [2], FISH (fluorescent in situ hybridization), and CGH (Comparative Genomic Hybridization) has facilitated the detection of submicroscopic chromosome aberrations. The molecular approach improves the etiological diagnostic of DD/ID, which is only achieved in around 30% of the evaluated cases [3]. Using this diagnostic method, we present molecular and clinical data of the first case of 8q22.2q22.3 microdeletion syndrome in Colombia.

This chromosome aberration is a clinically recognizable condition described worldwide in only seven patients [4–6]. The principal features of this syndrome are facial dysmorphism, ptosis, blepharophimosis, very short stature, developmental delay, and microcephaly. This report also lets us confirm the usefulness of molecular and cytogenetic analysis in the clinical approach of patients with idiopathic dysmorphism associated with DD/ID. The localization of the deleted genes and clinical phenotype are discussed.

## 2. Case Presentation

**Figure 1:**
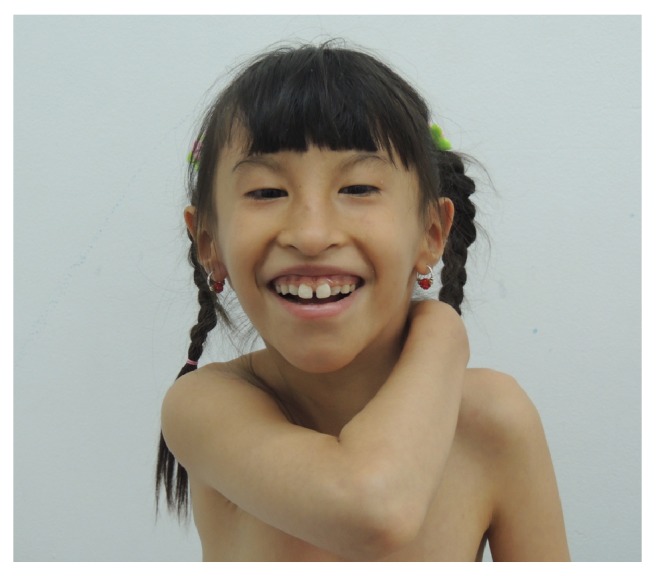
Microdeletion syndrome involving 8q22.2q22.3. Female patient at the age of 10 years with microcephaly, triangular face, arched eyebrows, blepharophimosis, bilateral epicanthic fold, oblique palpebral fissures, midface hypoplasia, nostrils hypoplasia, short philtrum, thin upper lip, and down-turned corners of the mouth.

The patient was a 10-year-old girl referred to our genetic department because of developmental delay, microcephaly, epilepsy, severe short stature, impaired speech, facial dysmorphism, congenital deafness, and skeletal abnormalities. She was the second of two born children of healthy nonconsanguineous parents and was born at 36 weeks due to intrauterine growth restriction and oligohydramnios, with a birth weight of 1,800 g and length of 42 cm (both bellow 2SD). She presented her first epileptic crisis at five months of age; it was characterized by generalized tonic-clonic seizures accompanied by eye deviation. By the age of 8 months, absent seizures began to present, and the generalized tonic-clonic seizures disappeared, although she has had careful follow-up by the neurologist, the pharmacological control of absence seizures has been partial. Her development has been severely delayed. She could sit at the age of 7 months; no crawling, independent standing was achieved at 30 months of age, and until the present, she persists with severe language development delay since she only recognizes names of few ordinary objects without having a fluent language. Facial dysmorphism was noted at one year of age; it consisted of a triangular face, blepharophimosis, telecanthus, epicanthus, palpebral ptosis, sparse eyebrows, low-set ears, flat malar region, thin upper lip vermillion, and down-turned corners of the mouth. At one year of age, she was diagnosed with bilateral hip dysplasia, conductive left hearing loss, and right mixed hearing loss. Currently, at the age of 10, she persists with the same facial dysmorphisms, severe short stature (110 cm -2SD), microcephaly (OFC 46 cm –2SD), small hands, short thumbs, bilateral fifth finger clinodactyly, severe cognitive impairment, absent of fluent speech, easy distractibility, and inadequate attention.  Figure 1 shows the phenotypic findings described in this case.

Sleep electroencephalogram shows only nonrapid eye movement (NREM) sleep associated with epileptogenic activity characterized by generalized high-voltage biphasic waves followed by slow and sharp waves (spikes) with right-sided predominance. She has a normal cerebral MRI, screening for inborn errors of metabolism was negative, and high-resolution conventional karyotype was normal 46, XX. According to the genome-wide array, CGH (Agilent Human Genome CGH Microarray 44K), a minimal/maximal deletion of 5.238 Mb and 5.374Mb, respectively, at 8q22.2q22.3 was diagnosed. The region contains 33 genes in a minimal interval of 99,509,806 to 104,747,354 and maximal interval of 99,509,429 to 104,883,122 (based on the March 2006 Human Genome Sequence Assembly [hg18]). The deletion was excluded in the mother's blood sample; the father was not available for investigation.

## 3. Discussion

Deletion of the 8q22.2q22.3 region has been associated with a clinically recognizable condition described worldwide in only seven patients. We present a new case comparing clinical and molecular data with previously reported cases. The deleted region comprises at least 25 clinical relevant genes, including the KCNS2, GRHL2, and COH1, that could be relevant in the pathogenesis of this syndrome.  Table 1 comprises all clinical data from patients previously reported and this new case. The ratio between male and female cases of 8q22.2q22.3 deletion syndrome seems to be skewed toward an excess of females (males: 2, females: 6). There is no evident aged-factor maternal or paternally related, and all cases are sporadic. Our patient had intrauterine growth restriction (IGR) diagnosed at the time of the delivery; it seems that IGR is not a common finding since seven out of the eight reported patients had uneventful pregnancy. Short stature and microcephaly are common findings in this condition, 3 out of 8 patients developed postnatal microcephaly, and 3 out of 8 had postnatal short stature.

All eight patients have a developmental delay of variable degrees; 4 out of 8 have a severe developmental delay, and developmental language delay seems to be a common factor in almost all the patients (Table 1). However, there is not a correlation between microcephaly, the size of the deletion, and severity of developmental delay. Our patient has a severe language developmental delay, probably worsened by her bilateral hearing loss. Our patient presents classical facial dysmorphism of an 8q22 deletion syndrome, including ptosis, blepharophimosis, epicanthus, thin upper lip vermillion, and down-turned corners of the mouth. There is no phenotype-genotype correlation for ptosis and blepharophimosis. However, there is a proposed critical region related to ptosis which has a protein-coding gene ZFHX4 (Zinc Finger Homeobox 4) in 8q21.11 [7], a gene that is out of the deleted area in the reported patients. It could play a positional or close regulation effect related to the deleted region.  Table 2 comprises molecular data (deleted region) from patients previously reported and this new case. Seizures and epilepsy are a remarkable diagnosis for almost all the cases since seven out eight patients have presented seizures.

Our patient has been affected by generalized tonic-clonic seizures and absence seizures since her first year of life. We do not have electroencephalographic details of the epileptic crisis of the others patients; however, according to the Kuechler et al.' report, one of the patients had absence seizures, and another one presented two epileptics crisis at six years old. In addition, Kuroda et al. [5] describe an 8-year-old patient, who has had difficult-to-manage seizures since age 10 months. Our patient has been very symptomatic all of her life, and her treatment had been difficult; she has been treated with multiple medicines. Nevertheless, she presents at least one absence seizure per day. Some of the genes in the deleted region are implied in seizures and epilepsy. For instance, KCNS2 (potassium voltage-gated channel, modifier subfamily S, and member 2) encodes a neuronal modulatory alpha subunit Kv9.2, associated with the transmission across chemical synapse, with the Dopamine-DARPP32 Feedback into the cAMP pathway, the regulation of the resting membrane potential, and the control of the shape and frequency of action potentials [8]. Kv.9.2 is highly expressed in the brain (olfactory bulb, cerebral cortex, the hippocampus, and the cerebellum) retina and spinal cord [8]. We could only hypothesize that epilepsy is somehow related to the absence of KCNS2 expression although it is not an entirely satisfactory explanation given the lack of retinal or cerebellar disease in the reported cases.

Our patient shares the majority of the clinical features described in the preceding report, including congenital hearing loss described in only one patient previously by Sinajon et al. [6] (Table 1). Interestingly, the GRHL2 gen (Grainyhead-like 2) at 8q22.3 [9] is associated with an autosomal dominant form of progressive no syndromic sensorineural hearing loss with a highly variable age of onset and progression. Homozygous GRHL2B mutant zebrafish embryos have enlarged otocysts, thinner otic epithelium, and smaller or eliminated otoliths [9].

Apparently, there is no relationship between this gene and epilepsy. However, loss of GRHL2 in zebrafish induces neural apoptosis and extinction of midbrain – hindbrain boundary (MHB) markers [10]. The VPS13B or COH1 gene (vacuolar protein sorting 13 homolog B (yeast)) is also located in the deleted region, Cohen syndrome is caused by mutations in the VPS13B gene [11], and it has an important role in eye, hematological, and nervous central system development. Despite the clinical variability of Cohen syndrome, our patient does not present phenotypic signs of Cohen syndrome, as truncal obesity, chorioretinal dystrophy, myopia, neutropenia, or classical facial dysmorphism. In only one of the previously reported cases, Cohen syndrome was suspected. However, VPS13B was not involved in that patient's deletion.

## Figures and Tables

**Table 1:** Clinical phenotype of the seven reported cases carrying an interstitial microdeletion of 8q22.2q22.3. Patient 8 is the new Colombian case reported.

	**Patient 1**	**Patient 2**	**Patient 3**	**Patient 4**	**Patient 5**	**Patient 6**	**Patient 7**	**Patient 8**
Sex	F	M	F	F	F	F	M	F

Age mother/father	20/21	24/30	38/36	35/31	n.d	n.d	22/25	38/37

Weeks of gestation	37	42	40	37	40	39 weeks and 4 days	41 or 42	36

Birth weight (g)	2,240 (-1.7 SD)	4,000 (+0.8 SD)	3,350 (-0.3 SD)	2,550 (-1 SD)	2,700 (-1.8 SD)	2,702 (-1.3 SD)	Over 3,600	1,800 (-2 SD)

Birth length (cm)	45 (-1.9 SD)	n.d	50 (-0.8 SD)	43.5 (-2.5 SD)	48 (-1.7 SD)	47.5 (-1.2 SD)	57 (+3.66 SD)	42 (-2 SD)

Birth OFC (cm)	32 (-1.4 SD)	36 (+0.1 SD)	34 (-0.7 SD)	31.5 (-1.7 SD)	33 (-1.5 SD)	33.5 (0.0 SD)	n.d	n.d

Age	6 years	3 years 6 months	8 years 6 months	8 years	20 years	8 years	40 years	10 years

Height (cm)	111 (-1.2 SD)	97 (-1 SD)	116 (-2.7 SD)	102.5 (-4.5 SD)	151	124.8 (-0.7 SD)	170.9	110 (-2 SD)

Weight (Kg)/BMI (kg/m2)	20 (-0.6 SD)/16.2	16.1 (+0.3 SD)/17.1	20 (-2 SD)/14.9	15.4 (-2.9 SD)/14.7	73.5/32	24.35 (-0.8 SD)/15.6	99/33.9	17 (-1.5 DS)/14

OFC (cm)	48.5 (-1.6 SD)	49 (-1.2 SD)	48.7 (-2.1 SD)	46 (-3.9 SD)	53 (-1.4 SD)	51.6 (+0.5 SD).	59.7 (+3.45 SD)	46 (-2 SD)

Developmental delay/ID	Severe	Moderate	Severe	Severe	Moderate	Moderate/severe	Moderate	Moderate

Sits independents (Months)	12	n.d	Between 24 and 36	24	9	7	n.d	7

Walks independent (Months)	27	17	60	90	24	24	13	30

First Words (Months)	No words	n.d	No words	No words	18-20	46	n.d	24

Seizures	+	-	+	+	+	++	+	++

Autistic behavior	+	+	-	+	+	+	+	-

Temper tantrums	n.d	+	-	+	+	n.d	+	-

Sleep disturbances	+	n.d	-	n.d	+	n.d	n.d	-

Low sensitivity to pain	+	n.d	-	n.d	n.d	n.d	n.d	-

Autoaggressiveness	+	+	-	n.d	+	n.d	+	-

Restlessness	+	+	-	n.d	+	n.d	n.d	+

Poor facial expression	+	+	+	+	-	+	-	-

Blepharophimosis	+	+	+	+	-	-	+	+

Telecanthus	+	+	-	+	-	+	-	+

Ptosis	-	-	+	+	-	-	+	+

Epicanthus	+	+	+	n.d	-	-	+	+

Eyebrows	Sparse, broad	Rather sparse	Rather sparse	Bushy/ Mild Synofris	Mild Synofris	Sparse and wide	Sparse	Sparse

Flat nasal tip	+	+	+	+	-	Slightly	-	+

Down-turned corners of the mouth	+	+	+	+	+	-	+	+

Ears	Small ear	Poor modeled ears	Poor modeled ears	Low set posteriorly rotated	Small ears	n.d	Overfolded and asymmetrical in length	Low set posteriorly rotated

Hands/Feet	n.d	Short thumbs and toes	Short hands with proximal implanted thumbs, mild cutaneous syndactyly bilateral proximal radio-ulnar synostosis	Single crease, bilateral clinodactyly of the 2nd and 5th fingers	Small hands with tapering fingers, II–III cutaneous syndactyly of feet	n.d	n.d	Short hands and feet, short thumbs, bilateral fifth finger clinodactyly

Congenital malformations	-	Large hiatal hernia, pylorus stenosis glandular hypospadias	n.d	Diaphragmatic hernia	-	-	Congenital heart disease. Hypoplastic auditory canals	-

Minimal deletion size (Mb)	5.26	6.10	5.26	6.44	1.92	1.36	3.35	5.23

+ denotes feature present; - denotes feature absent; n.d. denotes not documented.

ID: intellectual disability.

In conclusion patients with a submicroscopic deletion of 8q22.2q22.3 are characterized by a facial phenotype with blepharophimosis, telecanthus, epicanthus, flat malar region, thin upper lip vermillion, and down-turned corners of the mouth. Other clinical manifestations associated with 8q22.2q22.3 deletion syndrome can be a moderate to severe developmental delay, language development delay, absent speech, deafness, microcephaly, seizures, short postnatal stature, and congenital diaphragmatic hernias, especially when FPM2 (Zinc Finger Protein, FOG Family Member 2) is involved [4]. Until the present report, there is not a clear correlation between the size of the deletion and developmental delay, short stature, microcephaly, or seizures (Table 2).

**Table 2:** Summary of deletion data of patients 1–5 from Kuechler et al.; patient 6 from Kuroda et al.; and patient 7 from Sinajon et al. compared with the present report.

**Authors**	**Patients**	**First proximal deleted locus**	**Last distal deleted locus**	**Minimal deletion size (Mb)**
Kuechler et al.	Patient 1	99,895,352	105,151,384	5.26
	Patient 2	100,688,715	106,789,261	6.10
	Patient 3	99,293,796	104,556,255	5.26
	Patient 4	100,025,224	106,469,347	6.44
	Patient 5	102,017,156	103,939,573	1.92

Kuroda et al.	Patient 6	103,066,066	104,430,435	1.36

Sinajon et al.	Patient 7	100,142,380	103,492,901	3.35

Rincón et al.	Patient 8	99,509,806	104,747,354	5.23

Patients 1-5 and patient 8 based on UCSC Genome Browser hg18, March 2006, and patients 6 and 7 based on 2009 (GRCh37/hg19).

## Data Availability

No data were used to support this study.
